# New microbial resource: microbial diversity, function and dynamics in Chinese liquor starter

**DOI:** 10.1038/s41598-017-14968-8

**Published:** 2017-11-06

**Authors:** Yuhong Huang, Zhuolin Yi, Yanling Jin, Yonggui Zhao, Kaize He, Dayu Liu, Dong Zhao, Hui He, Huibo Luo, Wenxue Zhang, Yang Fang, Hai Zhao

**Affiliations:** 1 College of Pharmacy and Biological Engineering, Chengdu University, Chengdu, 610106 China; 2 Key Laboratory of Environmental and Applied Microbiology, Chengdu Institute of Biology, Chinese Academy of Sciences, Chengdu, 610041 China; 3 Environmental Microbiology Key Laboratory of Sichuan Province, Chengdu, 610041 China; 4 Wuliangye Group, 150 West Minjiang Rd., Yibin, 644007 China; 5 Moutai University, Renhuai, 564501 China; 6 Bioengineering College, Sichuan University of Science & Engineering, Zigong, 643000 China; 7 College of Light Industry, Textile and Food Engineering, Sichuan University, Chengdu, 610065 China; 8 School of Liquor-Making Engineering, Sichuan University Jinjiang College, Meishan, 620860 China

## Abstract

Traditional Chinese liquor (Baijiu) solid state fermentation technology has lasted for several thousand years. The microbial communities that enrich in liquor starter are important for fermentation. However, the microbial communities are still under-characterized. In this study, 454 pyrosequencing technology was applied to comprehensively analyze the microbial diversity, function and dynamics of two most-consumed liquor starters (Jiang- and Nong-flavor) during production. In total, 315 and 83 bacterial genera and 72 and 47 fungal genera were identified in Jiang- and Nong-flavor liquor starter, respectively. The relatively high diversity was observed when the temperature increased to 70 and 62 °C for Jiang- and Nong-flavor liquor starter, respectively. Some thermophilic fungi have already been isolated. Microbial communities that might contribute to ethanol fermentation, saccharification and flavor development were identified and shown to be core communities in correlation-based network analysis. The predictively functional profile of bacterial communities showed significant difference in energy, carbohydrate and amino acid metabolism and the degradation of aromatic compounds between the two kinds of liquor starters. Here we report these liquor starters as a new functionally microbial resource, which can be used for discovering thermophilic and aerobic enzymes and for food and feed preservation.

## Introduction

Chinese liquor (Baijiu), approximately 40-60% alcohol by volume, is one of the world’s four most popular distilled spirits along with whisky, brandy and vodka. Chinese liquor production accounts for more than one-third of all spirits consumed in the world according to the International Wine and Spirit Research Group. The unique and traditional Chinese solid simultaneous saccharification and fermentation (SSF) technology have existed for more than 4000 years^1,2^. Even more intriguing, microbial communities associated with environmentally-enriched for Chinese liquor fermentation was significantly different from those of most western spirit productions^3^. Chinese liquor is classified primarily by its distinctive flavor and taste^4^. Nong-flavor (NF, also called thick flavor and strong flavor) liquor accounts for more than 70% of the Chinese liquor market^4^. Meanwhile, the consumption of Jiang-flavor (JF, also called sauce flavor) liquor has increased in recent years according to the China National Food Industry Association. These two most-consumed and popular liquors are highly valued in Chinese culinary culture^4^.

**Figure 1:**
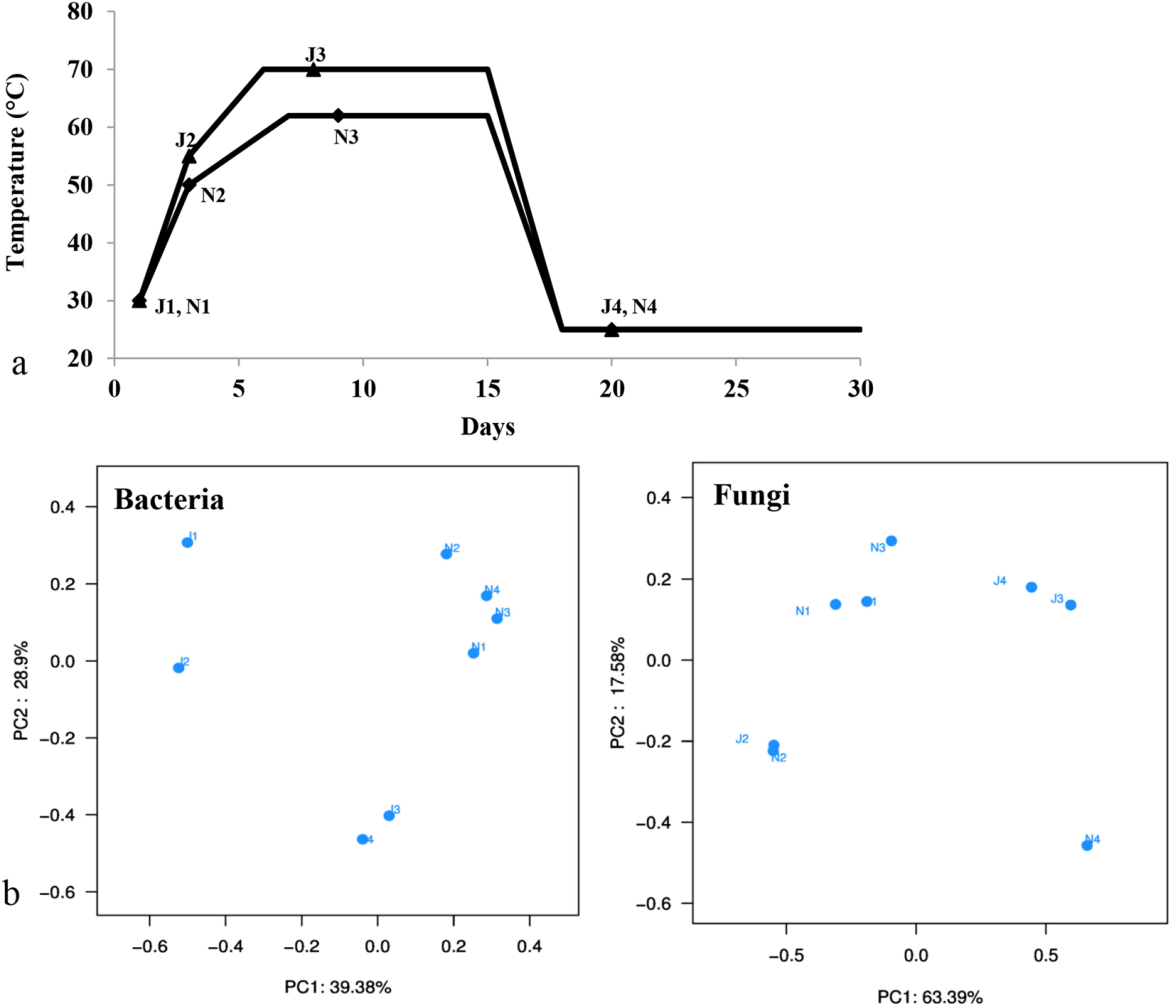
Differentiation in Jiang-flavor (J) and Nong-flavor (N) liquor starter samples. (**a**) Liquor starter samples were harvested at different time points. J1 and N1 were sampled at the beginning of liquor starter production; J2 and N2 were sampled after 3 days of liquor starter fermentation; J3 and N3 were sampled after 8 and 9 days of liquor starter fermentation, respectively; J4 and N4 were from the mature liquor starter. The temperatures of J1, J2, J3, J4, N1, N2, N3 and N4 were 30, 55, 70, 25, 30, 50, 62 and 25 °C, respectively. (**b**) Principal component analysis (PCA) of differentiation bacterial and fungal communities in the Jiang-flavor (J1-J4) and Nong-flavor (N1-N4) liquor starter samples. Each community was clustered using PCA.

The liquor SSF process is mainly attributed to the interaction and metabolism of microbes in liquor starter (daqu), zaopei and pit mud^5^. Among them, liquor starter is important for saccharification, fermentation and flavor-generation and is the most essential component for the production of Chinese liquor^6^. The production process for both NF and JF liquor starters usually takes approximately four months (Fig. 1a). Briefly, ground wheat is mixed with water and shaped into blocks, which are spontaneously fermented in a fermentation room for approximately one month and then dried in a storage room for another three months to mature^5^. During the one month period of fermentation, the room temperatures increase to 50 and 55 °C after three days, and quickly reach the highest values of 62 and 70 °C after around six and seven days for NF and JF liquor starters, respectively. This high temperature continues for approximately 7–8 days. After the high-temperature stage, the room temperature will finally decrease to approximately 25 °C. Both the NF and JF starters use wheat as feedstock, and no microorganisms are intentionally inoculated, as all the microbes are enriched from the feedstock, water, air and working environment. The main difference between the production processes of these two liquor starters is the changes of room temperature, which is mainly generated by microbial metabolic heat and is controlled by the placement of the starter blocks, turning over the blocks and ventilation. As NF liquor starter is characterized by moderately high temperature (62 °C), the blocks are usually tiled, spread and covered with a layer of straw in the fermentation room. The blocks must be turned every day to maintain the moderately high temperature for 8 days. However, JF is a high-temperature liquor starter (70 °C), so the blocks are heaped up and covered in thick straw. The blocks are only turned two or three times during the high-temperature period. Therefore, the dynamics of the microbial composition during the liquor starter production process are mainly determined by the working environment and the changes of room temperature during the liquor starter production process.

The liquor SSF process is a highly skilled and mature technology, and the microbial community in liquor starter is highly stable with a long history^7^. Nonetheless, the microbial diversity, function and dynamics of liquor starter, which are related to the dynamic changes of room temperature during production, have remained under-characterized. Microbial communities have been analyzed by traditional and molecular methods, e.g., culture-dependent methods^8–10^ and culture-independent PCR-DGGE^5,9–13^. However, many issues exist with these methods, such as missing most of the unculturable strains, time-consuming procedures, high workload, changing microbial composition, high cost, and, most importantly, a limited understanding of the microbial communities under study. Advances in high-throughput sequencing technology, such as 454 pyrosequencing, can comprehensively analyze the composition, diversity and richness of the microbial community for many samples in a single run^14^. Based on pyrosequencing analysis, the Fen liquor starter (also called qing flavor (QF) and light flavor) production process was found to have abundant microbes in the families Lactobacillaceae, Bacillacae and Saccharomycetaceae^15,16^. It was also shown that Firmicutes and Actinobacteria were the dominant bacteria in mature QF starter^17^. The dominant prokaryotic communities, including *Lactobacillus*, *Leuconostoc*, *Pseudomonas*, *Lactococcus* and *Thermoactinomyces*, were also detected in another type of mature NF liquor starter^18^. However, the liquor starter making process is complex, and the dynamics and interactions of the microbial communities in the above reported QF and NF liquor starters are still undercharacterized.

Co-occurrence analysis is a useful method to pinpoint the correlations and core modules in microbial communities^19–21^, and this method has not yet been applied in liquor starters. Recently, the software ‘Phylogenetic Investigation of Communities by Reconstruction of Unobserved States (PICRUSt)’^22^ was developed to predict the functional profiles of bacterial communities by bridging 16S rRNA gene information with a reference genomic database and the Kyoto Encyclopedia of Genes and Genomes (KEGG) database. Currently, this method can only be applied to predict bacterial function, and the number of available fungal genomes is not sufficient to allow for such prediction. To date, PICRUSt has been successfully applied to reveal the functional profiles of bacterial communities in animals^23,24^, humans^20,25^, oceans^26^, crude oil sediments^27^ and soil^28,29^.

In this study, the bacterial and fungal communities of two widely consumed liquor starters, NF and JF, were investigated using 454 pyrosequencing technology. Some thermophilic microbes were also isolated. Attempting to understand the comparative diversity, function and dynamics of microbial communities in liquor starter can help to improve Chinese liquor fermentation technology from “traditional experience-based model” to a “modern science-directed model”.

## Results

### Microbial diversity and richness in the liquor starters

Total bacterial 16S rRNA gene sequences (20765 and 51545) and fungal ITS sequences (54634 and 58912) were recovered from JF (J1, J2, J3 and J4) and NF (N1, N2, N3 and N4) liquor starter samples, respectively. The library samples were then clustered into bacterial and fungal Operational Taxonomic Units (OTUs) at 97% similarity (Table 1). After rarefied reads, the results showed that the most bacterial OTUs were found in the J3 and N2 samples (597 and 572 OTUs, respectively) and that the highest number of fungal OTUs in the J4 and N3 samples (587 and 258 OTUs, respectively) (Table 1). Across most of the samples, the OTU numbers of bacteria were much higher than those of fungi. The sequencing coverage for all the samples ranged up to 91% and was especially high for fungi at approximately 99%. A total of 315 bacterial and 72 fungal genera were found in the JF liquor starter. Meanwhile, 83 bacterial and 47 fungal genera were identified in the NF liquor starter. Samples N2 showed the highest values of Chao and Ace for 16S rRNA gene data, and N3 had the highest values of Chao and Ace for ITS data. Meanwhile, JF samples showed the highest values of Chao and Ace for 16S rRNA gene in sample J3 and the highest values of Chao and Ace for ITS in sample J4. The estimators of Shannon and Simpson also showed the similar results as the Chao and Ace indices discussed above.

**Table 1:** Community richness, diversity and coverage indices for Jiang-flavor (J) and Nong-flavor (N) liquor starter samples using 16S rRNA gene and ITS region rarefied to 3460 and 9622 sequences, respectively (OTU similarity was 97%). OTU: operational taxonomic unit. Higher values of Chao and Ace indicate more community richness.

	Sample ID	OTU	Ace	Chao	Coverage (%)	Shannon	Simpson
16S rRNA gene	J1	415	777	704	93.9	6.05	0.04
	J2	319	696	746	94.7	5.04	0.1004
	J3	597	1119	1087	91.0	6.59	0.0548
	J4	223	381	381	97.0	4.36	0.1363
	N1	342	742	689	94.2	4.07	0.1766
	N2	572	1321	1258	90.0	6.41	0.0411
	N3	478	987	974	92.1	5.84	0.0668
	N4	274	649	621	95.2	3.43	0.3014
ITS	J1	502	784	843	97.7	4.51	0.1555
	J2	210	302	311	99.2	1.98	0.6328
	J3	292	405	426	99.0	3.87	0.2964
	J4	587	853	892	97.7	6.13	0.0525
	N1	173	278	268	99.2	1.66	0.6484
	N2	138	213	205	99.4	1.04	0.806
	N3	258	388	372	98.9	2.96	0.2936
	N4	152	204	215	99.4	1.27	0.758

The Shannon and Simpson values indicate the community diversity. Higher Shannon values indicate greater community diversity, whereas a higher Simpson value indicates the opposite. The coverage value indicates the depth of sequencing. Bacteria (16S rRNA gene) and fungi (ITS) were analyzed in all the liquor starter samples. J1 and N1 were sampled at the beginning of liquor starter production; J2 and N2 were sampled after 3 days of liquor starter fermentation; J3 and N3 were sampled after 8 and 9 days liquor starter fermentation, respectively; J4 and N4 were sampled from the mature liquor starter. The temperatures of J1, J2, J3, J4, N1, N2, N3 and N4 were 30, 55, 70, 25, 30, 50, 62 and 25 °C, respectively.

Principal component analysis (PCA) was performed to comparatively analyze the relationships of the microbial communities in the JF and NF liquor starters. The bacterial community PCA scatterplot (Fig. 1b) revealed that the bacterial community in the JF liquor starter was distinct from that in the NF liquor starter. The bacterial community in the NF liquor starter was grouped to the right of the graph along PC1. However, the bacterial community in the JF liquor starter varied considerably. In contrast, the fungal community was similar in the JF and NF liquor starter samples during the early period (J1 and N1, J2 and N2) (Fig. 1b). However, the fungal community of liquor starter samples differed after the highest temperature period (J3, J4, N3 and N4).

### Microbial community composition

**Figure 2:**
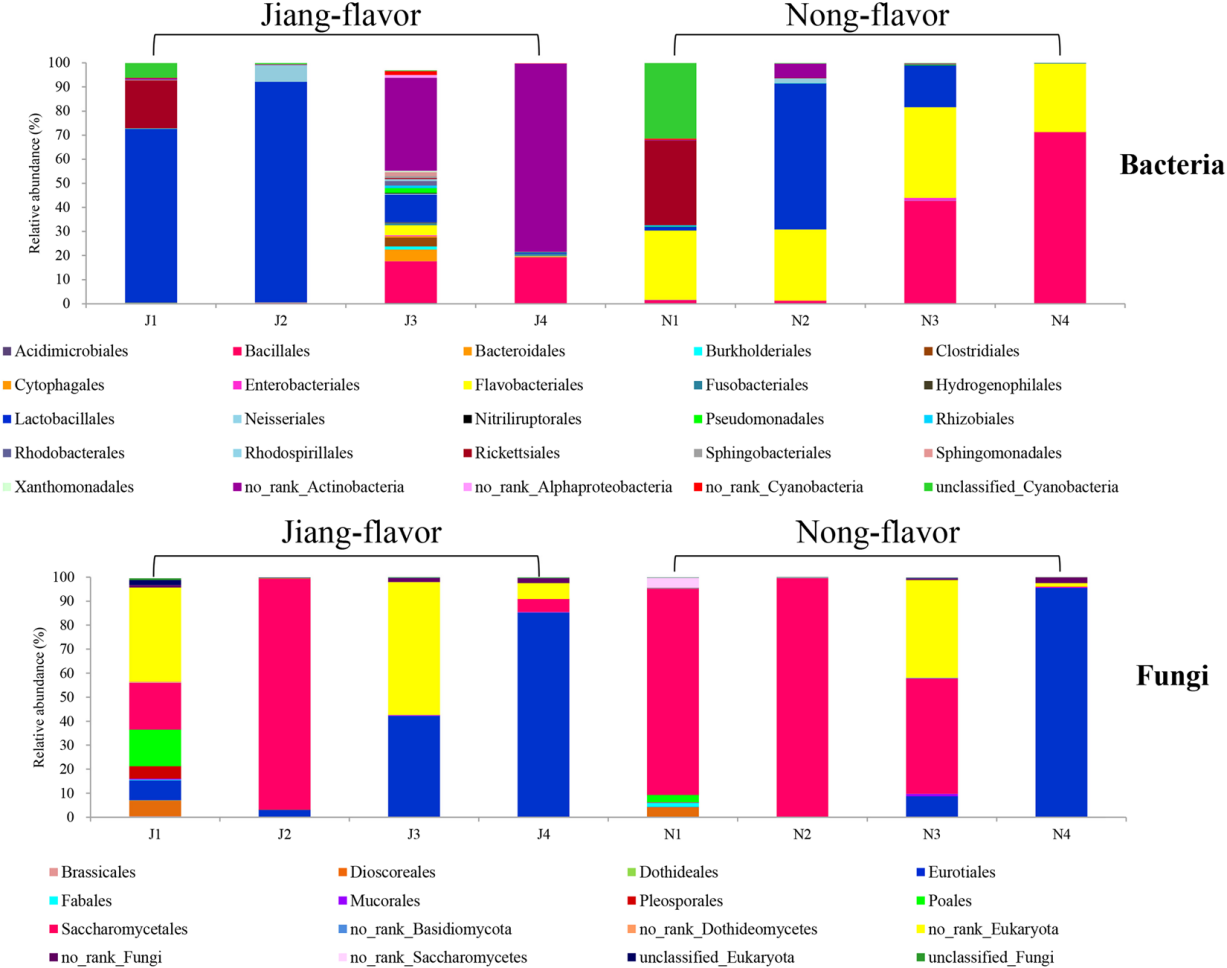
Microbial composition of the bacterial and fungal orders among the four Jiang-flavor (J1-J4) and four Nong-flavor (N1-N4) liquor starter samples (cutoff value: 0.2%). “Others” indicates rare or unclassified OTUs. J1 and N1 were sampled at the beginning of liquor starter production; J2 and N2 were sampled after 3 days of liquor starter fermentation; J3 and N3 were sampled after 8 and 9 days of liquor starter fermentation, respectively; J4 and N4 were from the mature liquor starter. The temperatures of J1, J2, J3, J4, N1, N2, N3 and N4 were 30, 55, 70, 25, 30, 50, 62 and 25 °C, respectively.

Classification of the bacteria and fungi at the order level for all samples is shown in Fig. 2. Lactobacillales increased up to 92% and 61% during the first 3 days for both J2 and N2 samples, respectively. After the temperature reached its maximum, Lactobacillales decreased markedly. However, Bacillales increased quickly and their abundance reached 17% and 43% for samples J3 and N3, respectively. Meanwhile, a relatively high abundance of no-rank Actinobacteria (39%) was found in J3, while only few (0.2%) were found in N3. Surprisingly, some relatively low abundance bacterial communities (see supplementary Table 1) were only detected in JF liquor starter. When the room temperature decreased to approximately 25 °C, Bacillales increased, especially in the NF liquor starter, reaching up to 71% (N4). No-rank Actinobacteria in the JF liquor starter also increased markedly, reaching 78% (J4). For bacterial communities, it was further shown that there was no OTU in common among four JF liquor starter samples (Fig. S1A), and 36 OTUs were in common among four NF samples (Fig. S1B). *Staphylococcus* spp. and *Chryseobacterium* spp. were the most abundant genera in common across the 4 NF liquor starter samples (data not shown).

The total fungal diversity in both the JF and NF liquor starters was much lower than that in the bacterial communities (Fig. 2). After 3 days of incubation, Saccharomycetales were the dominant fungi, accounting for up to 96.3% and 99.7% in samples J2 and N2, respectively. During the highest temperature period, a large number of no-rank Eukaryota were detected in both liquor starters, comprising 55% and 41% of the relative abundance in samples J3 and N3, respectively. In sample J3, Eurotiales also increased up to 42% during this period. Interestingly, 48% of Saccharomycetales could tolerate 62 °C and exist in sample N3. After the highest temperature period, Eurotiales increased markedly and became the dominant fungal taxon with up to 85% and 96% of the observed fungi in samples J4 and N4, respectively. For fungi communities, there were 10 OTUs (Fig. S1C) in common across four JF liquor starters. And 3 OTUs (Fig. S1D) were in common across the NF starters. The NF liquor starter had *Pichia kudriavzevii* and uncultured *Saccharomycetes* in common, while the JF liquor starter had *Pichia kudriavzevii*, *Thermomyces lanuginosus*, *Thermoascus crustaceus*, *Byssochlamys spectabilis*, *Rhizomucor pusillus*, *Emericella rugulosa*, *Eurotium* spp. and uncultured compost fungi species in common.

### Pivotal yeast, filamentous fungi and bacteria communities in liquor starter

In this study, five yeast genera, *Candida*, *Hyphopichia*, *Pichia*, *Saccharomycopsis* and *Saccharomycetes* were found in two liquor starters (Table 2). *Saccharomycopsis fibuligera* was detected in sample N1. However, *Saccharomycetes* were not found in the JF liquor starter, and a low abundance of no-rank/unclassified *Saccharomycetes* were present in the NF liquor starter. *Pichia* was the dominant genus and *P*. *kudriavzevii* was the most abundant species in samples J2 and N2. Recently, *P*. *kudriavzevii* and *Candida* spp. which can grow at temperatures up to 50 °C, have been isolated from NF liquor starter (N3). Surprisingly, *Hyphopichia*, which can tolerate higher temperature (62 °C), was relatively abundant (up to 40%) in the NF liquor starter (N3).

**Table 2:** Relative abundance of the main microbial community with ethanol fermentation capability.

Genus	Relative abundance(%)							
	J1	J2	J3	J4	N1	N2	N3	N4
*Hyphopichia*	0.09	0.11	0.01	0.00	83.03	0.11	40.40	0.00
*Pichia*	18.70	96.13	0.20	5.35	1.38	98.92	4.82	0.24
*Saccharomycopsis*	0.11	0.00	0.00	0.00	0.00	0.00	0.00	0.00
*Candida*	0.11	0.01	0.00	0.00	0.18	0.02	0.03	0.00
*Saccharomycetes*	0.00	0.00	0.00	0.00	5.30	0.10	0.10	0.10

J1 and N1 were sampled at the beginning of liquor starter production; J2 and N2 were sampled after 3 days of liquor starter fermentation; J3 and N3 were sampled after 8 and 9 days of liquor starter fermentation, respectively; J4 and N4 were from the mature liquor starter. The temperatures of J1, J2, J3, J4, N1, N2, N3 and N4 were 30, 55, 70, 25, 30, 50, 62 and 25 °C, respectively.

**Figure 3:**
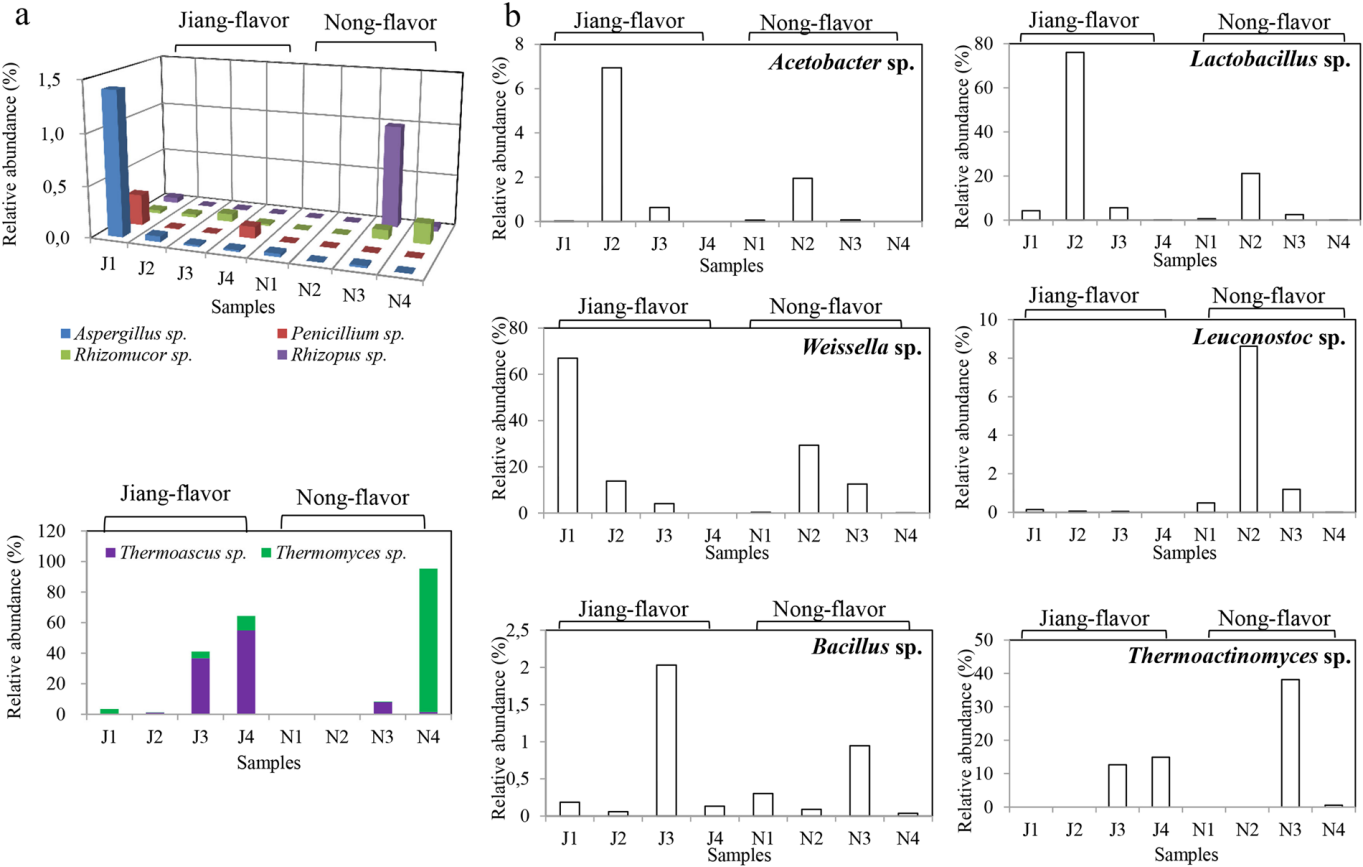
Relative abundance of the pivotal microbial communities in the Jiang-flavor and Nongs-flavor liquor starter samples. Main microbial communities are shown with saccharification capability (**a**) and with flavor generation capability (**b**).

When comparing the important filamentous fungi in the JF and NF liquor starters (Fig. 3a), both had low relative abundance of the traditional genera *Aspergillus*, *Penicillium*, *Rhizomucor* and *Rhizopus*. Interestingly, both liquor starters had high relative abundances of *Thermoascus* and *Thermomyces* at the highest temperature point (J3 and N3), and these increased even further when the temperature dropped to 25 °C (J4 and N4) (Fig. 3a). In the present work, some thermophilic fungi, *T*. *lanuginosus*, *T*. *crustaceus*, *R*. *pusillus*, and *R*. *microsporus* were successfully isolated from N3 and J3 samples.

For bacterial communities, similar abundances were found between *Acetobacter* and *Lactobacillus*, both of which increased quickly when the two liquor starters had incubated for 3 days (J2 and N2) and decreased when the temperature increased to its highest point (J3 and N3) (Fig. 3b). On the other hand, different abundances were shown between *Leuconostoc* and *Weissella* communities in NF and JF liquor starters (Fig. 3b). Both liquor starters had a relatively abundant *Bacillus* community during the highest temperature point (Fig. 3b, J3 and N3). Meanwhile, highly abundant no-rank *Actinobacteria* were found in JF liquor starter (J3 and J4) (Fig. 2). In addition, relatively abundant *Thermoactinomyces* bacteria were detected in N3 and J4, with comparable values in J3 and few found in N4 samples (Fig. 3b).

### Correlation Network analysis

A microbiome-wide network of microbial associations among 8 samples was performed to better understand the co-occurring relationship of the microbial community. In total, 339 and 239 pairs of nodes were significantly correlated in bacterial JF and NF samples, respectively (Table S2 and Table S3). Meanwhile, 69 and 68 pairs of nodes were strong correlated in fungal JF and NF samples, respectively (Table S2 and Table S3).

**Figure 4:**
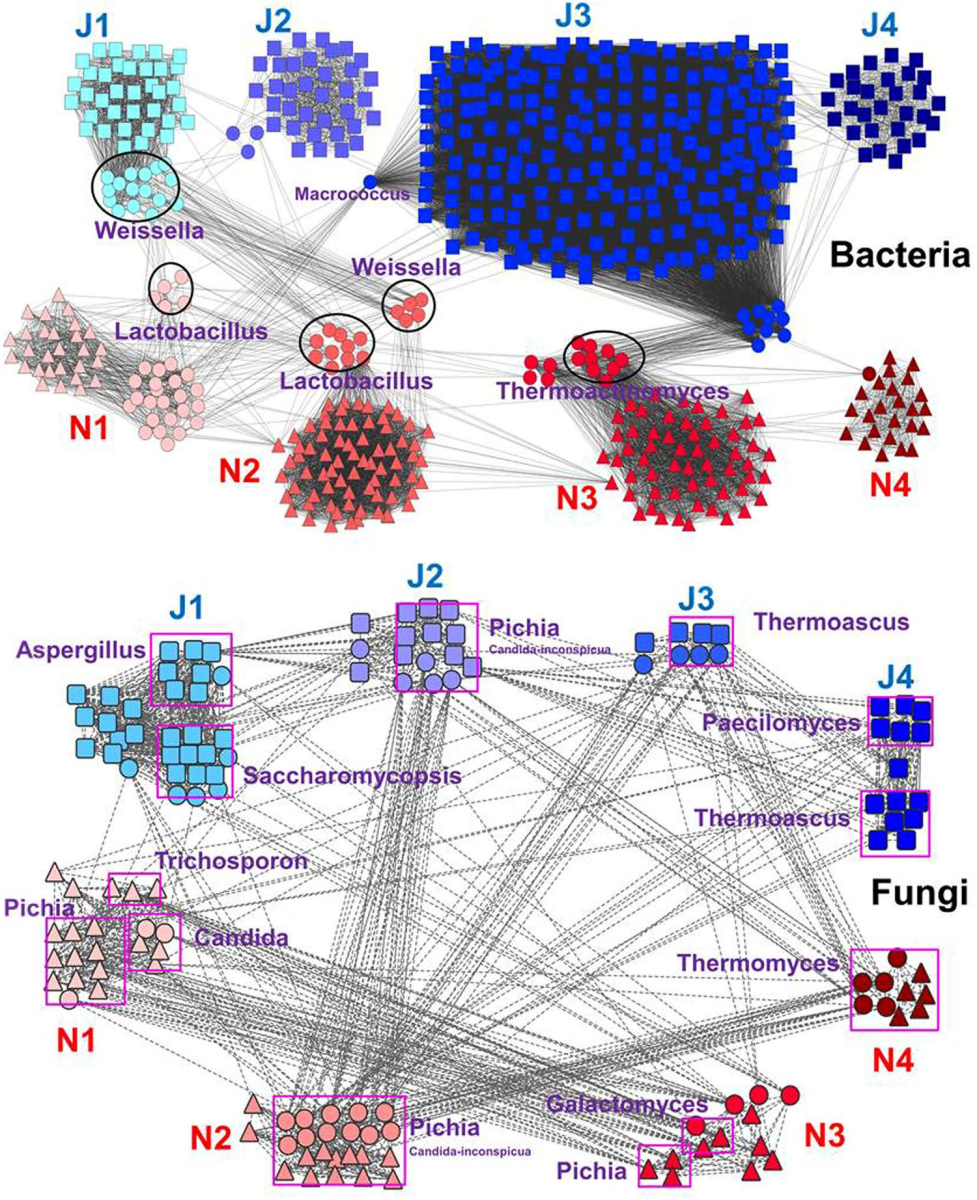
A correlation network of co-occurring microbial OTUs in the Jiang-flavor and Nong-flavor liquor starter samples. Each node represents a bacterial genus or fungal species, and color corresponds to different stages of liquor starter, with red for Nong-flavor (N1, N2, N3 and N4) and blue for Jiang-flavor (J1, J2, J3 and J4) samples. Square nodes stand for Jiang-flavor samples, triangle nodes stand for Nong-flavor samples, and circle nodes stand for co-occurring samples. Edges between nodes represent strong correlations (Spearman’s ρ > 0.7 for bacteria and ρ > 0.6 for fungi), including both positive and negative correlations.

In the bacterial network, hundreds of edges were found within each sample: 671 in N1, 2544 in N2, 1969 in N3, 289 in N4, 961 in J1, 544 in J2, 22983 in J3, and 438 in J4 (Fig. 4 and Table S3). Few edges were found among samples, however, except for N1-N2, N2-J1 and N3-J3, with 59, 71 and 90 edges, respectively (Fig. 4 and Table S2). Those associations among samples mainly resulted from co-occurring taxa. A large proportion of co-occurring taxa were found to be related to the genera *Weissella* (J1, N2), *Lactobacillus* (N1, N2), *Thermoactinomyces* (N3) and *Macrococcus* (J3) (Fig. 4 and Table S3).

Meanwhile, in the fungal network, the edge numbers within each sample were 153 (N1), 182(N2), 54 (N3), 28 (N4), 396 (J1), 90(J2), 26 (J3), and 88 (J4) (Fig. 4 and Table S4), which were all lower than those in the bacterial network. However, many associations were found among samples. Some associations even showed comparable edge numbers to those within samples, such as the 59 found in N1-N3, 24 in N2-N4, and 35 in N2-J2. Interestingly, the genus *Pichia* played a pivotal role in associations related to N1, N2, N3 and J2, accounting for 52% of nodes in N1, 92% of nodes in N2, 23% of nodes in N3 and 81% of nodes in J2, and contributing to 100% of the edges in N2-J2, N2-J3, N2-J4, N2-N4 and J2-J4 and 78% of edges in J2-N4 (Fig. 4, Table S4, Table S5). Additionally, the *Pichia* species were largely affiliated with *P*. *burtonii* (N1), *P*. *occidental* (N1) and *Candida inconspicua* (J2, N2 and N3). In addition to *Pichia*, the genera *Thermoascus*, *Paecilomyces*, *Thermomyces* also played important roles in fungal correlation network, accounting for 75% of nodes in J3 and 50% of nodes in J4, 43% of nodes in J4, and 100% of nodes in N4. Moreover, together with the genus *Pichia*, *Trichosporon* (N1), *Candida* (N1), *Galactomyces* (N3) and *Thermomyces* (N3) made large contributions to association between N1 and N3.

### Predictive functional profiles of bacterial communities

**Figure 5:**
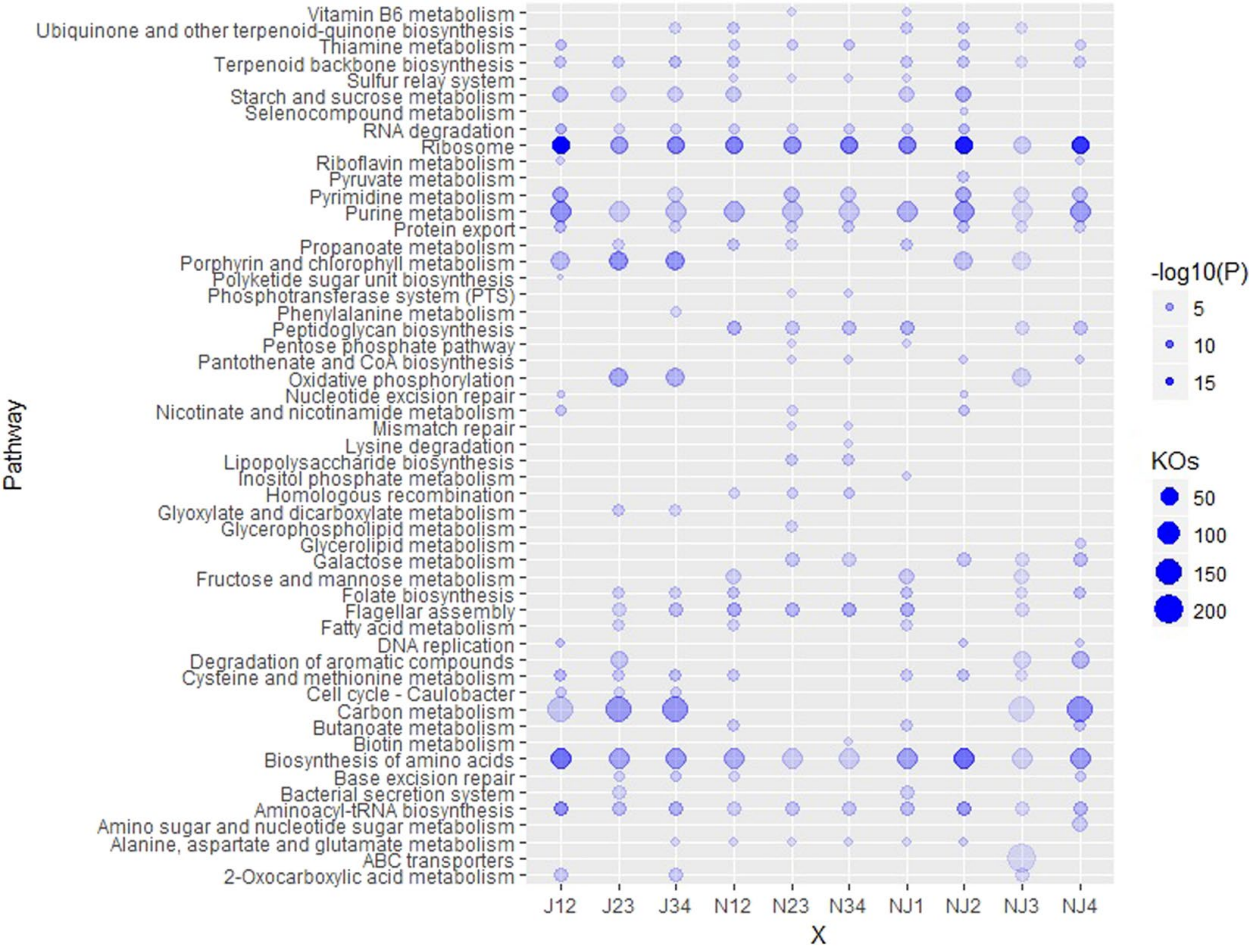
Differential abundancy in bacterial functional profiles among liquor starter. Differentially abundant KEGG pathways were analyzed between two samples from Nong-flavor and Jiang-flavor liquor starter samples, analyzing a total of 10 groups (J12, J1 and J2; J23, J2 and J3; J34, J3 and J4; N12, N1 and N2; N23, N2 and N3; N34, N3 and N4; NJ1, N1 and J1; NJ2, N2 and J2; NJ3, N3 and J3; NJ4, N4 and J4). The color in the bubble profile represent the differential significance of KEGG pathways (−Log_10_(Pvalue)) between two samples in every group, and the size of bubble stands for total gene number in each two-samples group. The top 20 differentially significant KEGG pathways in every group were applied to the construction of the bubble profile

Functional capacity of the bacterial communities during liquor starters making process was predicted by PICRUSt. Meanwhile the compositions of KEGG pathways were also analyzed for the 8 samples (Fig. S2). The abundant KEGG pathways were also analyzed between every pair of samples to investigate the differently functional profiles of bacterial communities among the 8 samples (Fig. 5). Notably, carbon metabolism was the most abundant pathways in 8 samples, especially for the microbes in the J3 sample. In addition to carbon metabolism, related carbohydrate and energy metabolism activities, such as oxidative phosphorylation, starch and sucrose metabolism, fructose and mannose metabolism, galactose metabolism, propanoate metabolism, sulfur metabolism, pyruvate metabolism, butanoate metabolism and glycolysis/gluconeogenesis, were highly represented in the top 50 pathways (Fig. S2). Interestingly, most of those carbohydrate and energy metabolism activities were in the top 20 significantly differential pathways in two-sample groups (Fig. 5), and some of them showed clear differences between two liquor starters. On the one hand, some pathways showed obviously higher differential significances compared to JF samples, e.g., starch and sucrose metabolism in J1-J2, J2-J3, J3-J4, N1-N2, N1-J1 and N2-J2, oxidative phosphorylation in J2-J3, J3-J4 and N3-J3, and carbon metabolism in J1-J2, J2-J3, J3-J4, N3-J3 and N4-J4 (Fig. 5). On the other hand, some pathways also showed higher differential significances compared to NF samples, e.g., N2-N3, N3-N4, N2-J2, N3-J3 and N4-J4 in galactose metabolism, and N1-N2, N1-J1 and N3-J3 in fructose and mannose metabolism (Fig. 5).

In addition, amino acid metabolism, terpenoid and polyketide metabolism were also found in the top 20 significantly differential pathways (Fig. 5). Among these, cysteine and methionine metabolism, lysine degradation and terpenoid backbone biosynthesis were in the top 30 pathways for 8 samples (Fig. S2). Importantly, cysteine and methionine metabolism (J1-J2, J2-J3, J3-J4, N1-N2, N1-J2, N2-J2 and N3-J3) and terpenoid backbone biosynthesis (J1-J2, J2-J3, J3-J4, N1-N2, N1-J2, N2-J2 and N3-J3) presented relatively high differential significances compared to JF samples. Furthermore, the number of genes involved in the degradation of aromatic compounds was much higher in J3 and J4 samples (Fig. S2) and showed highly differential significances in J2-J3, J3-N3 and J4-N4 (Fig. 5).

## Discussion

Understanding the diversity, function and dynamics of liquor starter microbial communities is important for improving liquor quality and yield. Although the unique and traditional Chinese solid state SSF and liquor brewing technology has continued for over several thousand years, the structure of the NF and JF liquor starter microbial communities is still not well characterized^5,8–13,15,17,18^. This is the first study to comprehensively analyze the microbial composition of Chinese NF and JF liquor starters during the production process. A total of 315 and 83 bacterial genera and 72 and 47 fungal genera were detected in the JF and NF liquor starters, respectively, indicating that both liquor starters have complex and abundant bacterial and fungal communities. This study obtained a relatively larger number of microbes and higher coverage than studies of Fen liquor starter (QF) and Maotai liquor starter (JF) which used traditional and molecular methods; fewer than 30 species was detected in one starter^5,8–13^. Moreover, our study found a surprising result that bacterial and fungal communities had the highest diversity and richness at the higher temperature stage in NF liquor starter. And in JF liquor starter, the highest diversity and richness of bacterial communities were also detected at the highest temperature stage of J3, while the highest diversity and richness of fungal communities were shown at the mature stage of J4.

The metabolic products of bacteria are important to develop unique flavors for liquor^4^. In this study, *Weissella*, *Lactobacillus* and *Thermoactinomyces* were identified as the most abundant bacterial taxa and made large contributions in the bacterial correlation network, so they could constitute the core genera in bacterial communities for these two liquor starters. The genera *Lactobacillus* and *Acetobacter*, belongs to the group of acetic acid bacteria. *Lactobacillus* is mainly applied to ferment traditional food and beverages^30^, and *Acetobacter* is the main functional bacteria for producing vinegar^31^. The metabolic products (e.g., acetic acid, lactic acid) of *Acetobacter* and *Lactobacillus* can be further synthesized by esterase into esters, such as ethyl acetate, ethyl lactate, isoamyl lactate, hexyl acetate, 2-phenylethyl acetate, 3-methylbutyl acetate and benzenacetate, which make large contributions to the special flavor of NF and JF liquors^32–34^. It has been reported that *Weissella* and *Leuconostoc* are usually present in fermented food along with lactic acid bacteria and are active as a good dietary source of vitamins^35^. Therefore, the *Lactobacillus*, *Acetobacter*, *Weissella* and *Leuconostoc* identified in both liquor starters may be important for flavor contribution. This study was the first discovery that JF liquor starter had a relatively high abundance of thermophilic Actinobacteria, which are an important and functionally diverse group of microbes with potential for biodegradation, carbon cycling and nutrient transformation^36,37^. Meanwhile, the highly abundant no-rank Actinobacteria in JF liquor starter (J3 and J4) could produce volatile organic compounds; the secondary metabolic products of *Actinomyces* could also contribute to flavor development^38^. *Thermoactinomyces* can produce plenty of thermostable extracellular proteolytic enzymes, e.g., alkaline proteinase^39^, glutamyl endopeptidase^40^ and carboxypeptidase^41^. This study further found that Bacillales and no-rank Actinobacteria were the dominant communities when the temperature increased to the highest point. *Bacillus* is well known to play an important role in liquor flavor generation^42,43^. It has been reported that *B*. *licheniformis* in JF liquor starter can produce specific flavor compounds such as 2,3-butanediol, 3-hydroxy-2-butanone, 2-methylpropionic acid and 3-methylbutanoic acid^44–46^. Interestingly, among bacterial community with relatively low abundant in JF liquor starter, *Burkholderiales* has a great ability to degrade a vast array of aromatic compounds^47^, and *Clostridiales* is an expert at reducing a wide range of cinnamic acid derivatives to their corresponding 3-phenylpropionic acid derivatives^48^. Additionally, *Fusobacteriales* and *Clostridiales* work as versatile bacteria and have unique abilities to ferment amino acids^49^. Furthermore, *Cytophagales* was reported to have a high capacity of degrading cellulose^50^. Surprisingly, *Lactobacillus*, *Leuconostoc*, *Lactococcus* and *Thermoactinomyces* were also dominant in the mature status of another NF liquor starter^18^.

Moreover, the predicted functional profiles of bacterial communities have performed for both liquor starters. The liquor starter samples in this study showed that the Nearest Sequenced Taxon Index (NSTI) values (0.007 to 0.062) (Table S6) was lower than the reported similar sample (soil) (0.17) in Langille’s work^22^, suggesting sufficient accuracy for metagenome predictions by PICRUSt. The prediction result indicated that both liquor starter samples had the most active energy metabolism, especially, J3 had the highest number of genes involved in almost all of the pathways (Fig. S2). This result also showed that the microbial community in sample J3 possibly had the most metabolic activity, which might be consistent with its highest temperature (70 °C). Those genes involved in carbohydrate digestion metabolisms were also high in both liquor starters. The two liquor starters might have preferences in carbohydrate digestion, e.g., starch and sucrose for JF samples, galactose, fructose and mannose for NF samples. Amino acid, terpenoid and polyketide metabolisms possibly were also active with differential significances between the two liquor starters (e.g., cysteine and methionine metabolism and terpenoid backbone biosynthesis in JF samples). Therefore, intermediates from those pathways (e.g., butanoate, protanoate, terpenoids, polyketides, aliphatic amino acids and branched-chain amino acids) might act as precursors or stimulators of the aromatic compounds in Chinese NF and JF liquor^33,34,45,51–54^. Additionally, the relatively high differential significance of cysteine and methionine metabolism in JF samples might be consistent with reports that cysteine and its metabolisms play an important role in the formation of JF compounds^46^. Furthermore, the degradation of aromatic compounds showed high differential significances in JF samples, and more genes related to this pathway were found in J3 and J4 when compared to NF samples and other JF samples by PICRUSt prediction, which is not surprising that many aromatic compounds and their derivatives have been found in JF liquor^32,45,54^. To some extent, the difference of those pathways between two liquor starters might be consistent with their different flavors. This observation might suggest a confirmation for difference in the flavor-development of those bacteria mentioned above, e.g., *Actinomyces* and *B*. *licheniformis* in JF liquor starter. In addition, the capacity of aromatic compounds degradation by *Burkholderiales* in JF liquor starter might be consistent with the PICRUSt predicted result regarding the pathways of degradation of aromatic compounds. In sum, these bacteria in these liquor starters could be significant for developing their special and well-characterized flavor of this liquor.

Yeast constitutes the most important microbial community in the ethanol production process^55^. As described above, the genus *Pichia* was the most abundant yeast and made large contribution in fungal correlation network, thus it could be one of the core genera in fungal communities in these two liquor starter. In Fen liquor starter (QF), *P*. *kudriavzevii* has been found to be the most abundant species^15,56^, which is similar to our study. The surprising discovery is that *Hyphopichia* could tolerate 62 °C. *Hyphopichia* has a broad habitat and unique ecological niches such as starch, rumen contents, food, insects and insect frass^57,58^. However, little is known about the thermostability of *Hyphopichia*. In addition to their function in the ethanol production, yeasts may also play a pivotal role in liquor flavor generation^59^. *P*. *anomala* has been reported to increase acetate ester-hydrolyzing esterase activity when mixed with *S*. *cerevisiae* in liquor fermentation^60^, and regulate several important volatile compounds in Chinese liquor, such as ethyl lactate, octanoic acid, and ethyl tetradecanoate^61^. When co-fermented with *S*. *cerevisiae*, *P*. *kluyveri* increased varietal thiol concentrations in Sauvignon Blanc wine^62^. Therefore, the yeast communities that are found in the NF and JF liquor starters may contribute to both ethanol fermentation and favor generation.

Filamentous fungi are significant during the production process of liquor starters for their saccharification function by secreting a wide spectrum of carbohydrate degrading enzymes^4^. *Aspergillus*, *Rhizomucor* and *Penicillium* have been well studied for their good lignocellulose hydrolysis performance^63–66^. Traditionally, *Rhizopus* and *Aspergillus* have been widely involved in fermenting food and beverages, and considered as the standards in saccharification^4,67,68^. Here, both *Rhizopus* and *Aspergillus* did not show high abundance throughout the production process. However, *Thermoascus* and *Thermomyces* were the most abundant fungi. Therefore, the important thermophilic fungi that were isolated from the NF and JF liquor starters were completely different from the fungi which were found by traditional notion and experience during Chinese liquor fermentation. In this study, *Thermoascus*, *Paecilomyces* and *Thermomyces* were identified as abundant fungal taxa and played a pivotal role in the fungal correlation network, so they could be the core genera in fungal communities. *T*. *aurantiacus* ^69,70^, *T*. *crustaceus* ^69^, *T*. *lanuginosus* ^71–73^ and *P*. *variotii* ^74,75^ have all been reported to be promising sources of thermophilic enzymes for high-level carbohydrate degradation. Currently, the microbiome in the giant panda gut^76^, wood-feeding higher termites^77–79^ and cow rumen^80^ have been closely studied, as they specialize in the efficient degradation of biomass. All these studies have underscored the importance of microbial communities in ecology. However, most of the dominant microbiomes are bacteria that live in anaerobic, high water content or animal temperature conditions. It has been reported that filamentous fungi are the most potent biomass degraders, as they produce a high number and a broad variety of enzymes^81^. Most studies on cellulase production have focused on aerobic species because the anaerobes show limited growth and do not produce enzymes in high quantities^82^. Furthermore, thermophilic enzymes have received significant attention for biomass degradation in industrial processes^83,84^. In this study, some thermophilic fungi have been further isolated and identified, e.g., *T*. *lanuginosus*, *T*. *crustaceus*, *R*. *pusillus* and *R*. *microspores*. Besides these isolated thermophilic fungi, more functional fungi will be isolated in future work from the promising microbial resource of liquor starter. These isolated fungi will be used for further discovering novel thermophilic enzymes.

This study provides insight into the diversity, function and dynamics of liquor starter microbial communities and sets the stage for understanding liquor starter microbial composition, paving the way for further active microbial community studies by metatranscriptomics and metaproteomics technologies and functional microorganism isolation, which may finally optimize the liquor production process, increasing alcohol yield and developing flavor generation. This new microbial resource can also be used for other industrial applications, such as bioenergy production, thermophilic and aerobic enzymes discovery, and fermented products used in food or feed preservation.

## Methods

### Liquor starter sampling

NF liquor starter was sampled from a fermentation workshop of Yibin Hongloumeng Distillery Group Co., Ltd in Yibing, Sichuan, China in July 2013. JF liquor starter was sampled from a fermentation workshop of Kweichow Hanwang Group Co., Ltd. in Renhuai, Guizhou, China in July 2013. Both liquor starters were sampled at different time points. Samples were harvested from three locations in the fermentation room at each time point. Samples J1 and N1 were collected at the beginning of liquor starter production; J2 and N2 were collected after 3 days of liquor starter fermentation; J3 and N3 were collected after 8 and 9 days of liquor starter fermentation (the second day after the temperature reached to the highest point), respectively; J4 and N4 were collected from the mature liquor starter. The temperatures of J1, J2, J3, J4, N1, N2, N3 and N4 were 30, 55, 70, 25, 30, 50, 62 and 25 °C, respectively (Fig. 1a). The liquor starter samples were frozen in liquid nitrogen immediately when harvested in the fermentation workshop, and samples were transferred to 50 ml RNase free Corning CentriStar™ centrifuge tubes (CORNING, 430828) and kept in dry ice. After all samples were harvested, they were transferred to Chengdu Biology Institute, Chinese Academy of Sciences on that day and stored in a -80 °C freezer for further research.

### Isolation of thermophilic fungi

A total of 10 g liquor starter samples (N3 or J3) was mixed with 90 ml sterile physiological saline (0.85% w/v sodium chloride) and homogenized for 30 min at 200 rpm at room temperature. Then, 0.2 ml of the diluted mixture (10^−1^, 10^−2^ and 10^−3^ dilution) was spread on both rose bengal chloramphenicol agar and dichlorane glycerol agar plates and incubated at 50 °C for 2–4 days. The genomic DNA of the isolates was extracted using the Biospin Fungus Genomics DNA Extraction Kit (BioFlux, Tokyo, Japan) according to the manufacturer. Approximately 10 ng of genomic DNA was used as the template for amplifying ITS sequences with I-5^TM^ 2X High-Fidelity Master Mix (Mclab, CA, USA) and a universal fungal primer set ITS4 (5′ TCCTCCGCTTATTGATATGC 3′) and ITS5 (5′ GGAAGTAAAAGTCGTAACAAGG 3′)^85^. The amplification was performed as follows: 94 °C for 5 min; 30 cycles at 94 °C for 30 s, 55 °C for 30 s, 72 °C for 90 s; and a final extension at 72 °C for 10 min.The ITS sequences were purified and sent to TsingKe (Chengdu, China) for sequencing. All ITS sequences were deposited in GenBank under the accession numbers in Table 3.

**Table 3:** Accession numbers of ITS sequence for the isolated thermophilic fungi.

Source	Microorganism	Sequence ID	Accession number
N3	*Rhizomucor pusillus*	BankIt1856062 seq 1	KT737214
N3	*Rhizopus microsporus* 1	BankIt1856062 seq 2	KT737215
N3	*Rhizopus microsporus* 2	BankIt1856062 seq 3	KT737216
N3	*Thermomyces lanuginosus*	BankIt1856062 seq 4	KT737217
N3	*Thermoascus crustaceus*	BankIt1856062 seq 5	KT737218
N3	*Pichia kudriavzevii*	BankIt1856062 seq 6	KT737219
N3	*Candida* spp.	BankIt1856062 seq 7	KT737220
J3	*Thermoascus crustaceus*	BankIt1856062 seq 8	KT737221
J3	*Thermomyces lanuginosus*	BankIt1856062 seq 9	KT737222
J3	*Rhizomucor pusillus* 1	BankIt1856062 seq 10	KT737223
J3	*Rhizomucor pusillus* 2	BankIt1856062 seq 11	KT737224

### Genomic DNA extraction and 454 pyrosequencing

Three liquor starter samples for each time point were mixed well and ground using liquid nitrogen and total genomic DNA was extracted from 1.0 g of liquor starter using the E.Z.N.A Soil DNA kit (D5625-02, Omega, USA). To amplify the 16S rRNA genes and ITS genes, a universal bacterial primer set 27 F (5′ AGAGTTTGATCCTGGCTCAG 3′) and 533 R (5′ TTACCGCGGCTGCTG GCAC 3′) and a universal fungal primer set ITS1 (5′ TCCGTAGGTGAACCT GCGG 3′) and ITS4 (5′ TCCTCCGCTTATTGATATGC 3′) were used. Approximately 10 ng of genomic DNA was used as the template in PCR mixtures (50 μl) with rTaq premix (TaKaRa) and 100 nM of each primer. The amplification in triplicate was performed as described above. The PCR products were checked using 2% agarose gel electrophoresis and harvested by using the AxyPrep DNA Gel Extraction Kit (Axygen, INC., AP-GX-250) and sent to Shanghai Majorbio Bio-Pharm Technology Co., Ltd. (Shanghai, China) for sequencing on the Roche GS FLX 454 pyrosequencing platform. Before sequencing, the PCR products were purified and checked according to the QuantiFluor-ST fluorescent quantitation System (Promega) using the PicoGreen dsDNA Quantitation Reagent. The PCR products were then pooled at equal concentrations based on a standard curve. Emulsion-based clonal amplification (EmPCR) products were obtained using Roche emPCRAmp*-*Lib_L Kit (Roche). The samples were further prepared according to the GS FLX + Sequencing Method Manual_XL + Kit and were submitted for 454 pyrosequencing (Roche 454 FLX+).

### Statistical analysis

The raw sequences were trimmed of the adaptor, barcode, forward and reverse primer using SEQCLN (14-03-20) (http://sourceforge.net/projects/seqclean/)^86^. The resulting sequences were then quality-filtered by MOTHUR software package (v.1.33.0)^87^ to remove low quality sequences (shorter than 200 bp, with quality scores < 25, containing any ambiguous bases (N) or homopolymers longer than 6 nucleotides). The valid sequences were reduced to a subset of unique sequences in order to eliminate redundancy and reduce computation time. The reduced sequences were then clustered into OTUs at the 97% similarity level.

In detail, for bacterial OTU clustering, chimeric sequences in reduced sequences were identified and removed by UCHIME (4.2.40) (http://drive5.com/uchime)^88^. Uncorrected pairwise distances were calculated according to sequences’ base differences. Then, sequences having 97% similarity were clustered into OTUs using the furthest neighbor method in MOTHUR.

Meanwhile, for fungal OTU clustering, the reduced sequences were blasted against the reference Silva eukarya database (silva SSU111) (http://www.arb-silva.de/)^89^ to check the reference-based chimera by using the script identify_chimeric_seqs.py (QIIME 1.7.0, Usearch61) (http://qiime.org/scripts/identify_chimeric_seqs.html)^90^, and all chimeric tags were removed. Then sequences having 97% similarity were clustered into OTUs using the script pick_otus.py (QIIME 1.7.0, Usearch61) (http://qiime.org/scripts/pick_otus.html)^90^.

The Ribosome Database Project (RDP) Naive Bayesian classifier (11.2) (http://rdp.cme.msu.edu/)^91^ in MOTHUR was used to analyze bacterial and fungal clustered OTUs against reference SSUrRNA (small subunit ribosomal RNA) database in Silva (silva SSU111)^89^ for taxonomical classification. To fairly compare all samples at the same sequencing depth, the number of the sequences was normalized by randomly extracting 3460 reads for 16S rRNA gene data and 9622 reads for ITS sequence data from each sample using daisychopper.pl^92^ before alpha diversity indices analysis. Alpha diversity indices of Chao1, ACE, Shannon, Simpson and sample coverage (Good’s coverage) were calculated using QIIME 1.7.0^90^. PCA was constructed using R package to quantify differences in community composition^93^. A Venn diagram was created with the online tool Venny (2.1) (http://bioinfogp.cnb.csic.es/tools/venny/). The sequencing data was deposited in the NCBI Sequence Read Archive database under the study accession number PRJNA283746.

### Correlation Network Analysis

All microbial OTUs in liquor starter samples were used for network analysis. Spearman’s rank correlations between selected OTUs were calculated using R package^93^. A valid co-occurrence was selected as a strong correlation if the Spearman’s correlation coefficient (ρ) was >0.6 and >0.7 for fungi and bacteria, respectively^94^. Correlation networks were constructed with the open source platform Cytoscape (3.3.0)^95^.

### Predictive functional profiles of bacterial communities involved in metabolic pathways

PICRUSt (1.0.0)^22^ and KEGG^96^ were applied to predict functional differences of bacterial communities in the liquor starter samples based on the raw 16S rRNA gene data. Briefly, the closed-reference OTU was chosen to blast against the Greengenes database (13.5)^97^, then the metagenomes of selected OTUs were predictively analyzed against the KEGG database. The prediction of PICRUSt was evaluated by the Nearest Sequenced Taxon Index (NSTI)^22^. Differentially abundant KEGG pathways were calculated using the program Metastats^98^ (using an FDR correction, p <  = 0.05, Log_2_(FC)| >  = 1), and pathways with significant difference were used for drawing bubble profile.

## Electronic supplementary material

Supplementary materials
