# Supplementary material for: New microbial resource: microbial diversity, function and dynamics in Chinese liquor starter
Source: Sci Rep. 2017 Nov 6;7:14577. doi: 10.1038/s41598-017-14968-8 (PMC5674051; doi:10.1038/s41598-017-14968-8)
Supplement: Supplementary file 1 — Supplementary materials [file 41598_2017_14968_MOESM1_ESM.pdf]

New microbial resource: microbial diversity, function and dynamics in Chinese liquor starter

Yuhong Huang[1,2,3#], Zhuolin Yi[2,3#], Yanling Jin[2,3], Yonggui Zhao[2,3], Kaize He[2,3], Dayu Liu[1], Dong Zhao[4], Hui He[5], Huibo Luo[6], Wenxue Zhang[7,8], Yang Fang[2,3*], Hai Zhao[1,2,3*]

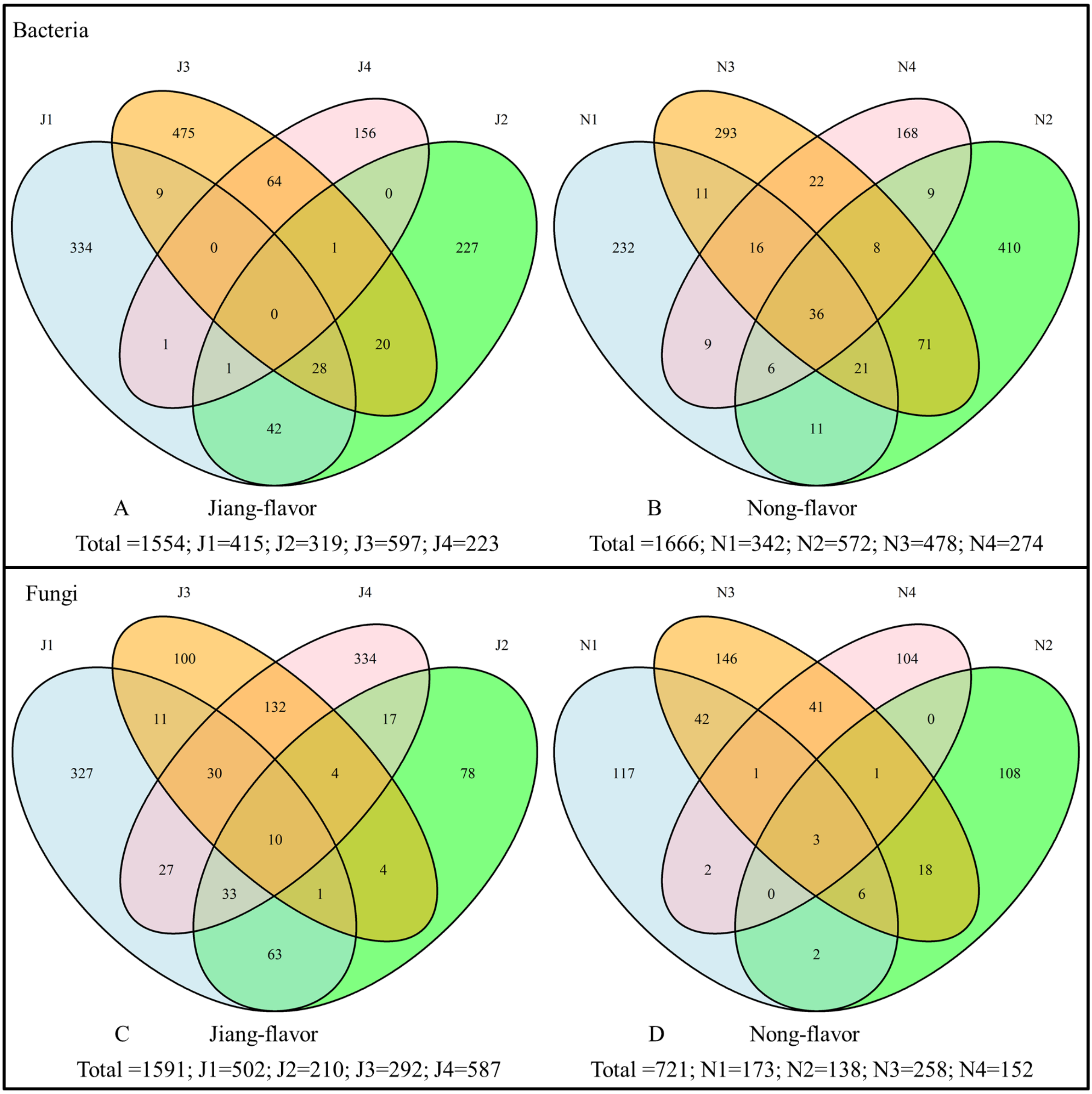

Fig S1 Venn diagram showing the unique and shared bacterial (above) and fungal (below) OTUs (97% identity level) in the Jiang-flavor (a and c) and Nong-flavor (b and d) liquor starter samples. Each sample is represented by one color, and the number of shared OTUs is in the overlapping area.

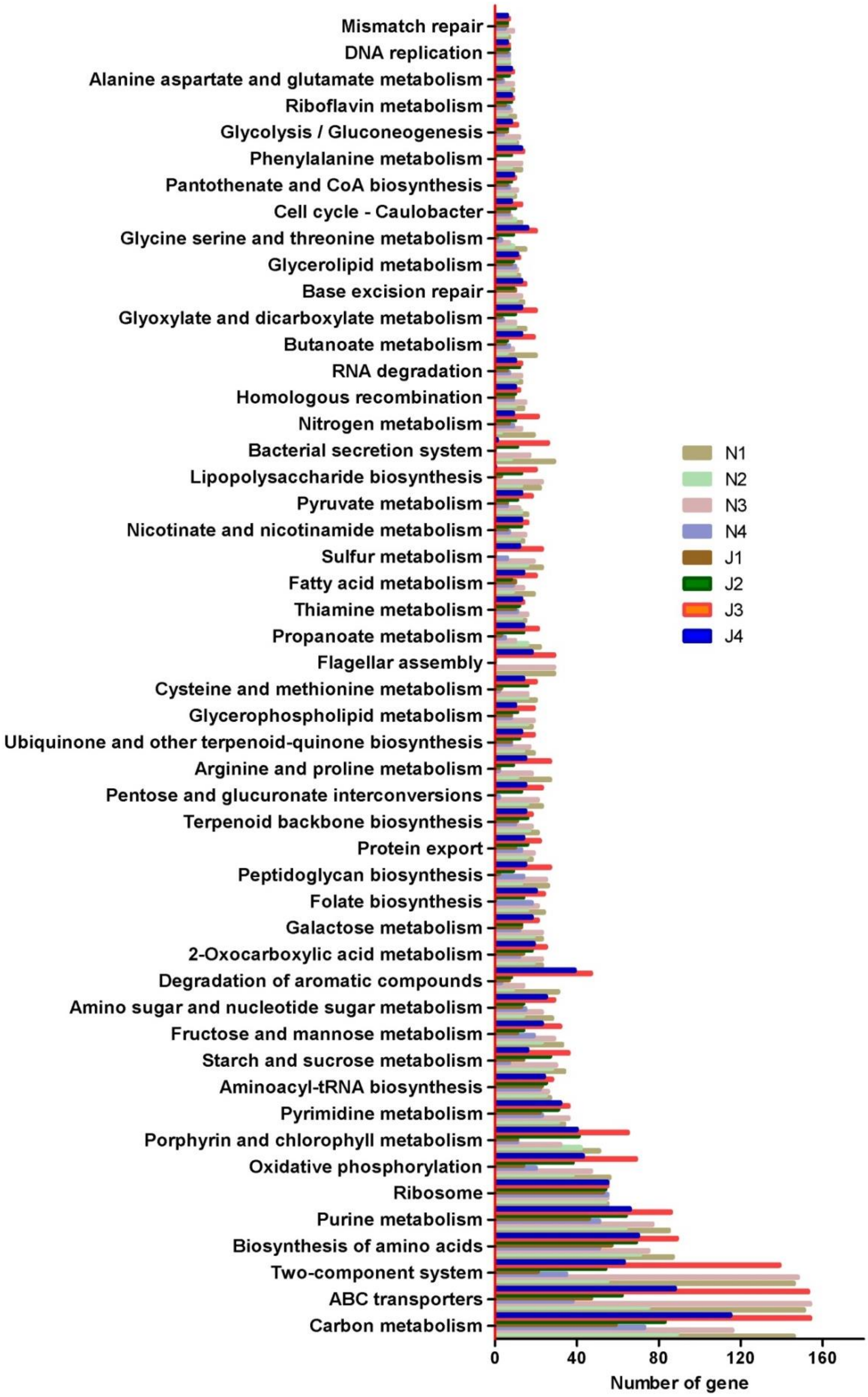

Figure S2. Bacterial functional profiles in the Nong-flavor and Jiang flavor liquor starter samples. The top 50 abundant KEGG pathways in the Nong-flavor (N1, N2, N3 and N4) and Jiang-flavor (J1, J2, J3 and J4) liquor starter samples was predicted by PICRUSt.

Table S1 Low abundant bacterial communities (order level) in Jiang flavor liquor starter

Bacteria	Abandance (%)
Acidimicrobiales	0.3
Bacteroidales	4.8
Burkholderiales	1.3
Clostridiales	3.7
Cytophagales	0.6
Fusobacteriales	0.8
Hydrogenophilales	0.4
Neisseriales	0.3
Nitriliruptorales	0.3
Rhodobacterales	2.0
Sphingobacteriales	0.7
Sphingobacteriales	0.7
Sphingomonadales	1.8
Xanthomonadales	0.5
no_rank Alphaproteobacteria	0.1

Table S2 Edges information in the bactarial correlation networks of Nong flavor (N1，N2，N3，N4) and Jiang flavor (J1，J2，J3，J4) liquor starter samples.

Edge numbers	N1	N2	N3	N4	J1	J2	J3	J4
N1	671	59	5	4	12	0	23	4
N2		2544	13	0	71	3	0	0
N3			1969	8	0	0	90	0
N4				289	1	0	0	0
J1					961	6	0	0
J2						544	0	0
J3							22938	26
J4								438

Table S3 BacteriaTaxonomic information of dominant modules in the correlation networks of Nong flavor (N1，N2，N3，N4) and Jiang flavor (J1，J2，J3，J4) liquor starter sample

	Number of nodes	Module hubs[#]		Abundant Genus
		All Bacteria	Co-exist Bacteria	
JX	339			
J1	52	Weissella(35), Lactobacillus(2), etc.	Weissella(16)	Weissella
J2	38	Lactobacillus(27), Weissella(4), Acetobacter(1), etc.	Lactobacillus(2), Acetobacter(1)	Lactobacillus, Weissella
J3	220	Bacillus(11), Sphingobacterium(9), Flavobacterium(8), Aciditerrimonas(7), Chryseobacterium(6), Clostridium-sensu-stricto(11), Ohtaekwangia(6), Corynebacterium(5), Dysgonomonas(5), etc.	Brevibacterium(1), Macrococcus(1), Delftia(1), Sphingobacterium(1), Wautersiella(1), etc.	Saccharopolyspora, Thermoactinomyces, Weissella, Lactobacillus
J4	29	Kroppenstedtia(13), Saccharopolyspora(4), etc.	-	Saccharopolyspora, Kroppenstedtia,
NX	239			
N1	56	Lactobacillus(9), Staphylococcus(8), Leuconostoc(4), Pseudomonas(3), Corynebacterium(3), Bacillus(3), etc.	Staphylococcus(3), Pseudomonas(3), Lactobacillus(4), etc.	Staphylococcus, Weissella,Leuconostoc, Lactobacillus
N2	88	Weissella (39), Lactobacillus(33), Acetobacter(6), Leuconostoc(6), etc.	Lactobacillus(9), Weissella(5), Lactococcus(1)	Weissella, Lactobacillus, Leuconostoc
N3	70	Thermoactinomyces(45),Weissella(3), Staphylococcus(3), etc	Thermoactinomyces(9), Cronobacter(1), etc.	Thermoactinomyces, Weissella, Staphylococcus
N4	25	Staphylococcus(25)	Staphylococcus(1)	Staphylococcus

[#]: the number of nodes for each microbe is indicated in brakets.

Table S4 Edges information in the fungal correlation networks of Nong flavor (N1，N2，N3，N4) and Jiang flavor (J1，J2，J3，J4) liquor starter samples.

Edge numbers	N1	N2	N3	N4	J1	J2	J3	J4
N1	153	6	59	2	3	2	4	5
N2		182	0	24	6	35	17	11
N3			54	0	9	1	3	0
N4				28	0	9	13	0
J1					396	17	0	0
J2						90	3	16
J3							26	10
J4								88

Table S5 Fungi Taxonomic information of dominant modules in the correlation networks of Nong flavor (N1，N2，N3，N4) and Jiang flavor (J1，J2，J3，J4) liquor starter samples.

	Number of nodes	Module hubs[#]	Abundant Genus
JF	69		
J1	31	Saccharomycopsis fibuligera(8), Eurotium amstelodami(3), Candida inconspicua(2), Aspergillus penicillioides(3), Aspergillus flavus(2), etc.	Saccharomycopsis, Pichia,
J2	16	Candida inconspicua(13), Cryptococcus neoformans(2), Hyphopichia burtonii(1).	Pichia
J3	8	Thermoascus crustaceus(6), Candida inconspicua(1), Emericella-nidulans(1)	Thermoascus, Thermomyces
J4	14	Paecilomyces variotii(6), Thermoascus crustaceus(5), Thermoascus aurantiacus(2), Thermomyces lanuginosus(1)	Paecilomyces, Thermoascus, Thermomyces, Pichia
NF	68		
N1	21	Pichia burtonii(7), Pichia occidentalis(4), Candida rugosa(2), Trichosporon jirovecii(2), ETC.	Hyphopichia
N2	24	Candida inconspicua(22), Pichia manshurica(1), Clavispora lusitaniae(1)	Pichia
N3	13	Galactomyces sp-TW_2011(3), Candida inconspicua(2), Thermoascus aurantiacus(2), Thermomyces lanuginosus(1), ETC.	Thermoascus, Hyphopichia,
N4	10	Thermomyces lanuginosus(10)	Thermomyces

[#]: the number of nodes for each microbe is indicated in brakets.

Table S6 The Nearest Sequenced Taxon Index (NSTI) value of eight liquor stater samples

Sample ID	NSTI
N1	0.029
N2	0.013
N3	0.048
N4	0.015
J1	0.007
J2	0.024
J3	0.062
J4	0.036
